# Cardiorespiratory Coordination in Hypercapnic Test Before and After High-Altitude Expedition

**DOI:** 10.3389/fphys.2021.673570

**Published:** 2021-05-24

**Authors:** Valentina V. Gultyaeva, Dmitriy Y. Uryumtsev, Margarita I. Zinchenko, Vladimir N. Melnikov, Natalia V. Balioz, Sergey G. Krivoschekov

**Affiliations:** Laboratory of Functional Reserves of Human Organism, Scientific Research Institute of Neurosciences and Medicine, Novosibirsk, Russia

**Keywords:** high altitude, mountaineers, cardiorespiratory coordination, hypercapnia, rebreathing, principal component analysis, exercise addiction

## Abstract

Coordination of cardiovascular and respiratory systems enables a wide range of human adaptation and depends upon the functional state of an individual organism. Hypoxia is known to elicit changes in oxygen and carbon dioxide sensitivity, while training alters cardiorespiratory coordination (CRC). The delayed effect of high altitude (HA) acclimatization on CRC in mountaineers remains unknown. The objective of this study was to compare CRC in acute hypercapnia in mountaineers before and after a HA expedition. Nine trained male mountaineers were investigated at sea level before (Pre-HA) and after a 20-day sojourn at altitudes of 4,000–7,000 m (Post-HA) in three states (Baseline, Hypercapnic Rebreathing, and Recovery). A principal component (PC) analysis was performed to evaluate the CRC. The number of mountaineers with one PC increased Post-HA (nine out of nine), compared to Pre-HA (five out of nine) [Chi-square (df = 1) = 5.14, *P* = 0.023]; the percentage of total variance explained by PC1 increased [Pre-HA median 65.6 (Q1 64.9/Q3 74.9), Post-HA 75.6 (73.3/77.9), *P* = 0.028]. Post-HA, the loadings of the expired fraction of O2, CO2, and ventilation onto PC1 did not change, and the loading of heart rate increased [Pre-HA 0.64 (0.45/0.68) and Post-HA 0.76 (0.65/0.82), *P* = 0.038]. During the Recovery, the percentage of total variance explained by PC1 was higher than during the Baseline. Post-HA, there was a high correlation between the Exercise addiction scores and the eigenvalues of PC1 (*r* = 0.9, *P* = 0.001). Thus, acute hypercapnic exposure reveals the Post-HA increase in cardiorespiratory coordination, which is highly related to the level of exercise addiction.

## Introduction

Ascent to high altitude (HA) is accompanied by the process of acclimatization to chronic hypoxia. Hypoxia is known to cause changes in the brain regulatory circuits, leading to alterations in blood flow and ventilatory sensitivity not only to oxygen but also to carbon dioxide (Lindsey et al., 2018). Six hours of hypoxic exposure is sufficient to lower the peripheral and central CO_2_ threshold (Richard et al., 2014). Moreover, ventilatory and cerebrovascular hypercapnic response patterns show similar plasticity in CO_2_ sensitivity following hypoxic acclimatization, suggesting an integrated control mechanism (Smith et al., 2017). Cerebral blood flow influences ventilation by altering central chemoreceptor stimulation *via* central CO_2_ washout at high altitudes (Hoiland et al., 2018). There are studies that describe the hematological (He et al., 2013) and hormonal changes (Liu et al., 2017), increased hypoxic tolerance, hypoxic ventilatory response and cerebral oxygenation (Cheung et al., 2014), and elevated sympathetic nervous system activity (Sander, 2016) after return to sea level. However, we are not aware of any studies that concern the delayed effects of HA acclimatization on the integration of cardiovascular and respiratory systems.

In general, the integration of the cardiovascular and respiratory systems can provide a wide range of adaptation of the human organism to varying environmental conditions and depends upon the individual functional state. The research approaches of modern physiology demonstrate the transition from the study of the organs and systems functioning to their interaction and integration, which help to improve prediction, particularly in sports physiology (Balagué et al., 2020). Cardiorespiratory coordination (CRC), based on the co-variation among cardiorespiratory variables is a new sensitive approach to studying intersystem integration. A principal component analysis of time series of cardiovascular and respiratory variables registered during cardiorespiratory exercise testing has been used for cardiorespiratory coordination determining to reveal training-specific physiological adaptations (Balagué et al., 2016; Garcia-Retortillo et al., 2019a) and manipulation-specific physiological state (Garcia-Retortillo et al., 2017, 2019b).

We have previously demonstrated that in swimming and skiing correlations between the responses of the cardiovascular and respiratory systems to acute hypoxic and hypercapnic tests are training-specific (Divert et al., 2015, 2017). Later we revealed that cardiorespiratory coherence changes in response to hypoxic exposure depend upon fitness status (Uryumtsev et al., 2020). The study of cardiorespiratory interactions is in its initial stage, and the influencing CRC factors are to be investigated. We suppose that acute hypercapnic exposure may reveal the peculiarities of coordination in the cardiorespiratory system due to the stimulation of central chemoreceptors in mountaineers several weeks after the return from HA.

In addition, we suggested that exercise addiction, which has been found in extreme sports (Heirene et al., 2016), could be correlated with cardiorespiratory coordination. Rock climbing athletes appear to experience withdrawal symptoms when they are abstinent from their sport comparable to individuals with substance and behavioral addictions (Heirene et al., 2016). Mountaineers ascent HA despite all the risks associated with this process. We assumed that the period between HA expeditions could be considered similar to the period of exercise deprivation in athletes with exercise addiction. Krivoschekov and Lushnikov (2017) described increased levels of anxiety and sympathetic nervous system activity assessed by Baevsky’s Stress Index (Baevsky and Chernikova, 2017) in athletes addicted to exercises during exercise deprivation (withdrawal states). Jerath et al. (2016) suggested that alteration in cardiorespiratory synchronization is the mechanism that underlies the symptoms of anxiety. Therefore, we expected that the higher the addiction score is, the worse the cardiorespiratory coordination would be during the period between HA expeditions.

Thus, the purpose of this study was threefold: (1) to compare the CRC in the hypercapnic test before and after the high-altitude expedition; (2) to compare the CRC before (at baseline) and right after the acute hypercapnia (during the recovery stage); and (3) to evaluate the correlation between the level of exercise addiction and CRC.

## Materials and Methods

### Subjects

The study included nine experienced healthy non-smoking male mountaineers aged 25–42 years. All subjects provided written informed consent prior to participation. The study protocol was approved by the Ethics Committee of the Scientific Research Institute of Neurosciences and Medicine (Novosibirsk) and performed in accordance with the Declaration of Helsinki.

### Procedure

The mountaineers were examined twice in June and September at an altitude of 164 m above sea level (Novosibirsk). The first round of investigations took place prior to ascending to a HA (Pre-HA). Then the subjects sojourned for 20 days in the mountains, living under the camp conditions at the altitude of 4,100 m with short-term ascents to the altitude of 6,500–7,000 m (Khan-Tengri, Tien Shan). The second investigation, similar to the first one, was performed on average 2 weeks (range 8–28 days) after descending the HA (Post-HA).

The mountaineers began the first visit by signing informed consent, measuring anthropometric characteristics, filling out the Exercise Addiction Inventory, and measuring office blood pressure. After a 30-minute rest including adaptation to breathing through a mask, we began recording gas exchange, ventilation, heart rate, and blood oxygen saturation. Physiological testing was performed in a sitting position through a facemask. All investigations were performed in the morning 2 h after a light breakfast by the same research assistant at an air temperature of 25°C in three functional states: breathing the ambient air (Baseline, 7 min of steady-state ventilation), hyperoxic hypercapnic rebreathing from a 5-L bag (Rebreathing, 3 min) and breathing the ambient air again (Recovery, 7 min). The entire measurement procedure hence took about 17 min. The Read modified rebreathing procedure was used to create hypercapnia (Read, 1967). An initial concentration of 5% CO_2_ and 40% O_2_ was created in the bag. To avoid the increased activity of peripheral chemoreceptors, the hyperoxic O_2_ concentration in the bag was maintained. The hyperoxic mixture was prepared using a NewLife (AirSep, United States) oxygen concentrator. The technique is described in detail elsewhere (Divert et al., 2015).

### Data Recording

A spiroergometric system Oxycon Pro (Erich Jaeger, Germany) was used for recording the following respiratory parameters: minute ventilation (VE), inspired and expired fraction of O_2_ (FiO_2_ and FeO_2_), inspired and expired fraction of CO_2_ (FiCO_2_ and FeCO_2_), and end-tidal O_2_ and CO_2_ partial pressure (P_ET_O_2_ and P_ET_CO_2_). Heart rate (HR) and blood oxygen saturation (SpO_2_) data were recorded by Pulse Oximeter BCI 3304 Autocorr (Smiths Medical, United States) and then automatically transferred to the Oxycon Pro. Office blood pressure was obtained by use of a sphygmomanometer (OMRON, Japan). We measured skeletal muscle mass by a multi-frequency tetrapolar bioelectrical impedance analysis device (InBody 370, Korea).

We used the Exercise Addiction Inventory (EAI) (Terry et al., 2004) to identify subjects at-risk from exercise addiction. The EAI consists of six statements based on a modified version of the components of behavioral addiction. Each statement has a five-point response option from “Strongly disagree” (1) to “Strongly agree” (5). The six statements that make up the inventory are: “Exercise is the most important thing in my life,” “Conflicts have arisen between me and my family and/or my partner about the amount of exercise I do,” “I use exercise as a way of changing my mood,” “Over time I have increased the amount of exercise I do in a day,” “If I have to miss an exercise session I feel moody and irritable,” and “If I cut down the amount of exercise I do, and then start again, I always end up exercising as often as I did before.” The EAI cut-off score for individuals considered at-risk of exercise addiction is 24.

### Data Analysis

Data analysis was carried out using the STATISTICA10 software package (StatSoft). To evaluate the effect of hypercapnia on the separate cardiorespiratory variables we averaged the data that we received during the last 2 min of the baseline and recovery periods, taking into account the low-frequency fluctuations of the cardiorespiratory variables with a period of about 2 min (Grishin et al., 2019). To describe a hypercapnic ventilatory response, we calculated CO_2_ sensitivity during rebreathing as the slope in the regression line of VE vs. P_ET_CO_2_ above the ventilatory threshold P_ET_CO_2_.

To study cardiorespiratory coordination, we used principal component (PC) analysis, which reflects the degree of coincidence of temporal patterns of physiological responses, that is, how much their increase and decrease are statistically synchronized. The total variance allows us to represent the time patterns of selected cardiorespiratory variables with fewer coordinating variables or PCs. The PC is extracted in descending order of importance. The number of PC reflects the dimension of the system, so a decrease in the number of PCs indicates greater coordination and vice versa. The PC number changes when the system undergoes reconfiguration. PC analysis was performed for each mountaineer on the time series of the following selected cardiorespiratory Pre- and Post-HA variables: VE, FeO_2_, FeCO_2_, and HR. Other recorded variables were excluded from the analysis due to their mathematical relationship with the above variables.

The number of PCs was determined by the Kaiser criterion, which considers a significant PC with eigenvalues ≥ 1.00. In the tables, we give the eigenvalues as a percentage of the total variance. The greater this percentage is, the greater the coordination of the variables projected onto PC appears. To analyze the effect of HA, the PC eigenvalues pre- and post- HA were compared over the entire 17-minute measurement. To find out the effect of hypercapnia on CRC, the PC eigenvalues for the time series of two states (Baseline and Recovery) were compared. A Wilcoxon Matched Pairs Test was performed to assess statistically significant differences in the cardiorespiratory variables, eigenvalues and PC loads Pre- vs. Post-HA and between the Baseline and Recovery states. The frequencies of occurrence were compared by the chi-square criterion. To test the suitability of the selected cardiorespiratory data for structure detection, Bartlett’s test of sphericity and the Kaiser-Meyer-Olkin Measure of sampling adequacy was used. The relationship between the level of exercise addiction and the eigenvalues of PC1 was evaluated by the Pearson correlation coefficient. To evaluate whether the Post-HA results were influenced by the delay following the high-altitude exposure, we calculated the Pearson correlation coefficient between the days of delay and the variables characterizing the CRC. Statistical significance was considered at a *P*-value < 0.05.

## Results

### Anthropometric Characteristics

The anthropometric descriptive characteristics of the subjects expressed as medians (Q1/Q3) are as follows: height 176 (171/180) cm, body weight 70 (65.7/83.1) kg, body mass index 23.8 (21.7/25.7) kg/m^2^, and muscle weight 34.6 (31.9/38.8) kg. The Exercise Addiction score was 18.0 (17/19) with a range 15–21.

### Effect of HA and Rebreathing on the Cardiorespiratory Parameters

Post-HA baseline blood pressure and recovery heart rate decreased significantly as compared to pre-HA (Table 1). There were no differences between studied respiratory variables as well as rebreathing data pre- and Post-HA. When comparing the variables at the recovery and the baseline stage, only one significant difference was found pre-HA. Recovery FeCO_2_ was lower than that at baseline, but the difference was less than 4%. Post-HA parameters at recovery and baseline were not different.

**TABLE 1:** Cardiorespiratory parameters and rebreathing test data pre- and post- high altitude.

Parameter	State	Pre-HA			Post-HA			P (Pre-post-HA)
		Me	(Q1/Q3)	*P* (1–2)	Me	(Q1/Q3)	*P* (1–2)	
VE (L min^–1^)	1 Baseline	10.5	(10.0/12.7)	NS	9.4	(9.1/10.0)	NS	NS
	2 Recovery	11.8	(7.6/12.8)		9.7	(8.6/10.7)		NS
FeO_2_(%)	1 Baseline	17.1	(16.6/17.2)	NS	17.0	(16.5/17.2)	NS	NS
	2 Recovery	17.0	(16.8/17.4)		17.1	(16.7/17.3)		NS
FeCO_2_ (%)	1 Baseline	3.83	(3.41/4.23)	0.028	3.69	(3.52/3.75)	NS	NS
	2 Recovery	3.69	(3.16/4.08)		3.62	(3.36/3.82)		NS
HR (beats min^–1^)	1 Baseline	69.2	(60.7/71.8)	NS	61.2	(56.4/62.3)	NS	NS
	2 Recovery	68.9	(62.0/74.4)		61.1	(55.3/63.5)		0.021
SBP	Baseline	128	(120/130)	–	117	(107/123)		0.042
DBP	Baseline	76	(66/86)	–	71	(60/76)		0.018
**Hypercapnic ventilatory response**								
P_ET_CO_2_ threshold (kPa)	Rebreathing	6.1	(6.0/6.2)	–	6.1	(5.8/6.1)	–	NS
Sensitivity (L min^–1^ kPa^–1^)	Rebreathing	16.2	(13.2/18.6)	–	21.8	(17.9/23.5)	–	NS

**VE, ventilation; HR, heart rate; HA, High altitude; Sensitivity, hypercapnic ventilatory sensitivity; NS, non-significant; P, Wilcoxon Matched Pairs Test.**

### Principal Component Analysis

Bartlett’s test of sphericity (*p* < 0.01) and the Kaiser-Meyer-Olkin Measure of sampling adequacy (min 0.53, max 0.74) showed the suitability of the selected cardiorespiratory data for structure detection. The PC analysis of the entire Pre-HA measurement revealed five mountaineers with one PC and four mountaineers with two PCs. Post-HA, the number of mountaineers with one PC significantly increased to nine [Chi-square (df = 1) = 5.14, *p* = 0.023]. There were no participants with two PCs Post-HA. Since we used the Kaiser criterion to determine the number of PCs in the model, the following PC (PC2, etc.) had eigenvalues < 1, i.e., they explained less variance than the original variables. Since one PC includes coordinated variables, one can conclude that the coordination of cardio-respiratory variables in the hypercapnic test increases Post-HA.

Post-HA, the percentage of total variance explained by PC1 significantly increased (Table 2). This also confirms an increase in cardiorespiratory coordination.

**TABLE 2:** Percentage of total variance explained by PC1 and PC2 pre- and post-high altitude.

	State	Pre HA			Post HA			P (Pre-Post HA)
		Me	(Q1/Q3)	*P* (1–2)	Me	(Q1/Q3)	P (1–2)	
PC1	Entire measurement	65.6	(64.9/74.9)	–	75.6	(73.3/77.9)	–	0.028
	1 Baseline	51.8	(49.0/54.3)	0.021	49.2	(47.1/53.7)	0.015	NS
	2 Recovery	67.0	(59.2/69.2)		62.2	(60.2/65.5)		NS
	Rebreathing	77.4	(75.8/86.2)	–	78.9	(72.7/88.2)	–	NS
PC2	Entire measurement	24.5	(20.6/26.3)	–	17.6	(13.8/19.3)	–	0.008
	1 Baseline	30.7	(25.8/33.2)	NS	32.3	(28.8/33.0)	NS	NS
	2 Recovery	23.8	(22.1/26.2)		24.6	(21.4/26.7)		NS
	Rebreathing	19.3	(11.7/22.6)	–	20.4	(10.0/26.0)	–	NS

**PC, principal component; HA, High altitude; NS, non-significant; P, Wilcoxon Matched Pairs Test.**

The loadings of VE, FeO_2_, and FeCO_2_ onto PC1 did not change Post-HA, and the heart rate loading significantly increased Post-HA (Table 3). This indicates that the coordination of respiratory variables with heart rate increased. Pre-HA, VE, FeO_2_, and FeCO_2_ formed PC1, whereas HR was involved in forming PC2 in four mountaineers with two PCs. Post-HA, in all nine mountaineers, all the selected variables (VE, FeO_2_, FeCO_2_, and HR) formed PC1.

**TABLE 3:** Projection of the cardiorespiratory variables onto PC1 pre- and post-HA (entire measurement).

	Pre-HA		Post-HA		
	Me	(Q1/Q3)	Me	(Q1/Q3)	P
VE	0.85	(0.84/0.89)	0.87	(0.84/0.90)	NS
FeO_2_	0.95	(0.93/0.95)	0.94	(0.94/0.97)	NS
FeCO_2_	0.91	(0.88/0.96)	0.91	(0.88/0.93)	NS
HR	0.64	(0.45/0.68)	0.76	(0.65/0.82)	0.038

**VE, ventilation; HR, heart rate; HA, High altitude; NS, non-significant; P, Wilcoxon Matched Pairs Test.**

The entire measurement segmentation by the states Baseline, Rebreathing, and Recovery revealed the maximal percentage of total variance explained by PC1 during Rebreathing (Table 2). During the Recovery stage, this percentage was significantly higher than that during the Baseline state. We did not find any significant differences in these percentages before and after the HA expedition.

### Relation of Exercise Addiction and Percentage of Total Variance Explained by PC1

**FIGURE 1:**
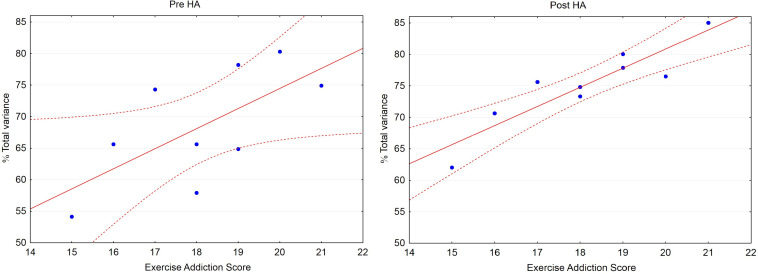
Scatterplot: Percentage of total variance explained by PC1 (% Total variance) vs. Exercise Addiction Score (EAS). Pre-HA: Correlation: *r* = 0.67 (*p* = 0.049), % Total variance = 10.79 + 3.18 • EAS. Post-HA: *r* = 0.90 (*p* = 0.001), % Total variance = 20.03 + 3.04 • EAS. Dashed lines depict 95% confidence interval.

Pre-HA, the Pearson correlation coefficients (*r*) between the exercise addiction score and the percentage of total variance explained by PC1 was 0.67 (*p* = 0.049), Post-HA *r* = 0.90 (*p* = 0.001) (Figure 1). The slopes in the regression equations both pre-HA and Post-HA were about 3. The exercise addiction score as well as the percentage of total variance explained by PC1 Pre- and Post-HA did not correlate with age (*r* = 0.31, 0.32, 0.31, respectively; NS), weight (*r* = −0.23, 0.01, −0.33; NS), BMI (*r* = −0.20, −0.22, −0.25; NS), and muscle mass (*r* = −0.11, −0.02, −0.26; NS).

### Correlation Between the Delay Following the HA Expedition and the Variables Characterizing the CRC Changes

There was a negative correlation between Post- and Pre-HA difference in the percentage of total variance explained by PC1 (Δ% Total variance) on the one hand and the delay following the high-altitude exposure during Rebreathing on the other hand (*r* = −0.86; *p* = 0.003; regression: Δ% Total variance = 16.9–1.3 ^∗^ Days Post-HA). The regression line crosses the zero level (i.e., CRC return to the Pre-HA value) on the 13th day. We did not find any significant correlation between Δ% Total variance and the delay following the HA exposure during Baseline (*r* = −0.02; NS), Recovery (*r* = 0.51; NS) and Entire measurement (*r* = −0.01; NS). There were no significant correlations between the loadings of VE, HR, FeO_2_, FeCO_2_ onto PC1 and the delay after HA expedition (*r* = 0.37, 0.10, 0.16, 0.30, respectively; NS).

## Discussion

Coordination among subsystems is a hallmark of physiologic state and function (Ivanov et al., 2016). Gas exchange, which is the outcome of a more or less coordinated work of the cardiovascular and respiratory systems, is regulated with the participation of the central and peripheral chemoreceptors that respond to the changes in the blood O_2_ and CO_2_ concentration as well as the pH in the intercellular fluid. The peculiarities of the cardiorespiratory coordination largely determine the individual reserves and physical ability (Balagué et al., 2016; Garcia-Retortillo et al., 2019a). They are of particular importance for mountaineers. Mountaineering is associated with hypoxic high-altitude sojourn, cold, and intense physical activity. The present study was aimed at studying the delayed effect of high-altitude exposure on the peculiarities of cardiorespiratory coordination revealed during central chemoreceptors stimulation. To the best of our knowledge, the results of this pilot study show for the first time an increase of cardiorespiratory coordination revealed by hypercapnic exposure in mountaineers several weeks after the high-altitude expedition. The second result concerns the increase in CRC during the recovery period after acute hypercapnia, which indicates a coordinating effect of acute hypercapnia. The third result demonstrates a high correlation between the exercise addiction scores and the CRC values after the high-altitude expedition.

The value of the ventilation threshold to CO_2_ in our study coincides with the value of the central chemoreflex recruitment threshold for ventilation in healthy young men of 46 mmHg (Keir et al., 2019). Previously, researchers considered the features of the influence of hypercapnia on ventilation, using the concepts of threshold, response, reactivity, and sensitivity (slope). This approach has shown some specific ventilatory responses to hypercapnia and hypoxia possibly because of adaptation to frequent high-altitude climbing (Puthon et al., 2016) or training-specific physiological adaptation (Divert et al., 2017), genetic factors (Goldberg et al., 2017), and even the presence of patent foramen ovale (Davis et al., 2019). We have not found any differences in the considered respiratory parameters at rest, as well as ventilatory thresholds and CO_2_ sensitivities before and after the high-altitude expedition. Probably, the delayed effect of altitude acclimatization on the studied respiratory parameters varies greatly between individuals. At the same time, heart rate and blood pressure significantly reduced after the expedition. According to He (He et al., 2013) the entire process of deacclimatization may take ≥ 100 days.

We revealed the remaining effect of acclimatization on cardiorespiratory coordination several weeks after return from high altitude. Using the principal component analysis, we were able to analyze the cardiorespiratory coordination over the entire measurement, including the transition periods between the baseline, rebreathing, and recovery states. This approach allows obtaining additional information about the CRC, even with the respect to non-stationary states. Various indices obtained as a result of the PC analysis for the entire 17-minute measurement showed the CRC increase Post-HA: the increase in the number of mountaineers with one PC (all the subjects), the increase in the percentage of variance explained by PC1 (eigenvalues), and an increase in the heart rate loading onto PC1. This is consistent with the results obtained by the same method, which has shown the higher sensitivity and responsiveness of cardiorespiratory coordination to exercise effects compared to isolated cardiorespiratory outcomes (Balagué et al., 2016; Garcia-Retortillo et al., 2017, 2019a). The last studies were performed during physical exercise testing. Increasing metabolic demand can be the coordinating factor for cardiorespiratory variables during exercise. In mountaineers Post-HA, changes in cardiorespiratory coordination are presumably associated with the plasticity of medullar regulatory circuits in response to long-term hypoxic stimulation of chemoreceptors (Lindsey et al., 2018).

The negative correlation between the Post- and Pre-HA difference in the percentage of total variance explained by PC1 and days Post-HA during Rebreathing indicates that the Post-HA rebreathing CRC was influenced by the delay following the high-altitude exposure. Meanwhile, we did not find any relationship between Post- and Pre-HA difference in CRC and days Post-HA during Baseline, Recovery, and Entire measurement. Thus, on the one hand, the large variability in the Post-HA exposure re-evaluation delay is a limitation of the study, but on the other hand, this allowed us to estimate the duration of the effects of HA acclimatization on the CRC after returning to the sea level.

The second result concerns an increase in CRC during the recovery period after acute hypercapnia, indicating an acute coordinating effect of hypercapnia. It has long been known that ventilation begins to increase linearly when the concentration of CO_2_ increases above the ventilatory threshold (Read, 1967; Duffin, 1990). This fact is used in the rebreathing test. However, we found for the first time that in the period immediately after the rebreathing, the percentage of the total variance, explaining PC1, also increases compared to the baseline level. In addition to ventilation and FeCO_2_, FeO_2_, and heart rate are included in the PC in our study. It should be mentioned that in our CRC study, we do not concern another type of cardiorespiratory coupling, respiratory sinus arrhythmia (RSA). However, hypercapnia is known to increase the amplitude of RSA (Tzeng et al., 2007; Brown et al., 2014), which also indicates an increase in cardiorespiratory coupling.

The third result relates the cardiorespiratory coordination to the exercise addiction score. When we included the EAI in our methods, we expected to identify subjects at-risk of exercise addiction similar to those found in extreme sports (Heirene et al., 2016). We assumed that the higher the addiction score is, the worse the cardiorespiratory coordination would be during the period between HA expeditions. However, the results obtained turned out to be the opposite. There was a high positive correlation between CRC and EAI scores. Moreover, none of the investigated climbers has reached the cut-off score of 24 points to be at-risk of exercise addiction. All examined subjects had a medium EAI score. Therefore, it is not possible to talk about withdrawal states. In our study, the level of exercise addiction as well as CRC did not correlate with age, weight, BMI, and muscle mass. An increase in the exercise addiction score indicates a greater commitment to a training lifestyle. We could assume that the EAI score is a psychological correlate of the fitness level in mountaineers. Therefore, the obtained high positive correlation between exercise addiction scores and CRC could be explained by the interdependence of these indices on the level of fitness. This is confirmed by the fact that it is after the high-altitude expedition that there is a high correlation. The concept of fitness for mountaineers includes the adaptation of the chemoreflex regulation circuit, as well as physical ability. However, this assumption requires further verification.

The practical relevance of the results obtained is that the cardiorespiratory coordination could serve as an additional marker of specific physiological adaptations to different hypoxic states, including high-altitude hypoxia in mountaineers and the pathological state accompanied by hypoxia.

### Limitations

The main limitation of the study is the lack of blood pressure time series, which could significantly improve the PC model. Another limitation is the lack of blood and biochemical analyses, including changes in pH, and indices of autonomic nervous system regulation, which could provide a more detailed integration of dynamics across different systems. Large variability in the Post-HA exposure re-evaluation delay could be a limitation of the study, but this allowed us to estimate the duration of the effects of HA acclimatization on the CRC after returning to the sea level. Moreover, this study investigates nine subjects only, which may limit the statistical power of the study.

### Further Considerations

In the future, we consider it appropriate to investigate the effect of autonomic nervous regulation on cardiorespiratory coordination.

### Conclusion

Acute hypercapnic exposure reveals the Post-HA increase in cardiorespiratory coordination, which is highly related to the level of exercise addiction. Acute hypercapnia provides a coordinating effect on the cardiorespiratory system. The CRC after the acute hypercapnia during the recovery stage is significantly higher than at baseline. These facts require further investigation.

## Data Availability Statement

The raw data supporting the conclusions of this article will be made available by the authors, without undue reservation.

## Ethics Statement

The studies involving human participants were reviewed and approved by Ethics Committee of the Scientific Research Institute of Neurosciences and Medicine (Novosibirsk). The patients/participants provided their written informed consent to participate in this study.

## Author Contributions

SK: conceptualized the research question, study design, and supervised the entire project. DU: performed the data analysis. VG and MZ: drafted the manuscript. VM and NB: collected data. All authors interpreted the results and critically reviewed and significantly contributed to the manuscript and approved the final version.

## Conflict of Interest

The authors declare that the research was conducted in the absence of any commercial or financial relationships that could be construed as a potential conflict of interest.
